# Performance of the BD-FACS Presto for CD4 count and hemoglobin measurement in a district hospital and rural laboratory in Ghana

**DOI:** 10.1371/journal.pone.0212684

**Published:** 2019-02-22

**Authors:** Zelda Moran, Jilian A. Sacks, Francis Kwabena Frimpong, Atta Boahen Frimpong, Yanis Ben Amor

**Affiliations:** 1 Earth Institute, Columbia University, New York, New York, United States of America; 2 Millennium Promise Alliance, Accra, Ghana; Brighton and Sussex Medical School, SOUTH AFRICA

## Abstract

**Introduction:**

In Ghana, initiation of Antiretroviral Therapy (ART) is recommended for all patients with an HIV diagnosis, regardless of CD4+ T-cell count. However, measurement of CD4 count remains an important metric for identifying patients with advanced HIV disease, and assessing a person’s overall immune status, which informs the decision to offer opportunistic infection screening and prophylaxis. Access to CD4+ T cell count in rural health facilities remains a major challenge in Ghana and other resource-limited settings. This study aimed to validate the accuracy of the BD FACSPresto near-patient device for measurement of CD4 count and hemoglobin concentration against the FACSCount (CD4) and Sysmex (hemoglobin) diagnostic machines when operated in both a district hospital and rural laboratory, serving a network of health posts in Ashanti Region, Ghana.

**Methodology:**

In the first phase of the study, patients were recruited from a district hospital, and both venous and capillary blood samples were tested using the FACSCount and Sysmex as reference tests and compared to results of the FACSPresto performed in the clinic laboratory at the district hospital. In the second phase, patients were recruited from both the hospital and from rural health clinics, and samples were tested using the FACSPresto at a rural laboratory. Sensitivity and specificity among samples categorized into different clinically relevant CD4 count ranges were calculated, along with correlation between the Presto and the reference measurements, and mean and relative bias with limits of agreement.

**Results:**

The FACSPresto was successfully operated in both clinical settings. A total of 59 samples in the first phase and 48 samples in the second phase were included. Positive bias was observed when comparing CD4 count measured by BD FACSPresto to FACSCount in the district hospital (bias = 44, LOA -72,160) and in the rural laboratory setting (bias = 74, LOA -96, 244). In addition, capillary blood samples were shown to give higher measures when compared to venous blood samples from the same participant. All results were statistically significant (p<0.05) apart from hemoglobin measurement in venous blood in the rural laboratory. Correlation coefficients were high for CD4 count measures and lower for hemoglobin measures.

**Conclusion:**

Overall, the Presto gave higher estimates of CD4 count compared to FACSCount, and hemoglobin measurements were higher than from Sysmex. Samples of capillary blood in turn gave higher results for both measurements compared to venous blood, consistent with previous analyses. These findings should be considered when selecting CD4 count machines for use at the point of care, especially in remote areas where capillary blood sampling may be preferable, but are likely balanced by device’s ease of use, portability, and ability to expand access to services. These results are some of the first to demonstrate the accuracy of the FACSPresto in West Africa and show that this device can be successfully operated in a very rural lab setting and may therefore assist to provide CD4 count and hemoglobin concentration measurement to populations in need.

## Introduction

Proper clinical management of people living with HIV (PLHIV) requires access to laboratory testing, including CD4 enumeration, liver and renal function tests and viral load (VL) monitoring. Since 2015, the World Health Organization (WHO) has recommended that antiretroviral treatment (ART) be initiated as soon as possible after diagnosis with HIV, regardless of CD4 count[1], and if viral load testing is available, CD4 is no longer needed for long-term monitoring for stable patients [2]. However, despite this “treat all” recommendation, CD4 testing remains important for establishing baseline immune function and identifying patients with advanced HIV disease (defined as CD4 T-cell count below 200 cells/μl), who are at higher risk for opportunistic infections (OIs) [1, 3]. In addition, CD4 count can be an important indicator of treatment failure [2]. Patients who are unstable or have advanced disease should receive a package of services, including screening and prophylactic treatment for certain OIs, or may need to be referred to specialized care. Therefore, it remains important that PLHIV on ART, especially those beginning ART for the first time or after a treatment gap, have access to CD4 enumeration.

Access to advanced diagnostic equipment for CD4 and viral load testing can be limited in rural or remote communities, and patients may need to travel long distances for testing. Point-of-care (POC) diagnostic tests, which are those used at or near the patient and outside of a laboratory, can greatly increase access to services when lab capacity is limited. POC testing for HIV infection has been widely adopted, and POC CD4 count testing shows potential for improving disease management in many settings. A 2016 systematic review found that POC CD4 testing is generally acceptable and feasible in low and middle income countries, and overall, prompt CD4 count testing upon diagnosis with HIV has been shown to improve rates of linkage to care, and initiation of ART [4, 5]. Therefore, POC testing can facilitate access to services and higher quality of care, in both rural and urban areas. Introducing well-validated devices for HIV diagnosis and monitoring is crucial to ensuring that all PLHIV have appropriate care, and given the number of CD4 measurement devices on the market, device-specific studies are needed to inform the selection of technologies for different settings [6].

There are numerous CD4 testing machines on the market, which vary greatly in sample throughput and size, ranging from high-volume devices such as the BD FACSCalibur and FACSCount, to smaller devices such as the CyFlow Mini POC (Sysmex), Pima (Abbott), and CD4NOW (PointCare), which also provides hematology. Wide ranges of bias have been reported across devices, highlighting the importance of using the same technology for longitudinal patient monitoring whenever possible [7]. For the purpose of regulatory approvals, the Presto has been validated against the FACSCalibur as reference standard for CD4 enumeration [8–10], but FACSCount is also widely used, with instruments reported in 48 countries and accounting for 31.6% of total reported CD4 instruments in a 2013 survey of WHO member states [11]. In comparison, FACSCalibur was reported in 33 countries, and 12.5% of total CD4 instruments [11].

The BD FACSPresto (Becton Dickinson, San Jose, CA, USA)—referred to here as Presto—is a portable, near-patient device which weighs less than 7 kg and able to be run using battery-power [12, 13]. The device was released in 2014 and provides absolute CD4 count, percentage of CD4 T-cells out of total lymphocytes, and hemoglobin (Hgb) concentration in a single test, with a total test time of approximately 25 minutes. Hgb concentration is a measurement of anemia, which is particularly relevant for pregnant women attending antenatal care (ANC). Therefore, having on-site, quick access to CD4 enumeration and Hgb measurement in one test could be important for prevention of mother to child transmission (PMTCT) of HIV programs. It has received regulatory approval by the US-FDA, the European Union (CE-IVD) and the WHO Prequalification (PQ) Programme, among others, in which its performance was compared to FACSCalibur [12, 14].

The Presto has been primarily studied in sub-Saharan Africa [8, 14–21] and is designed for use in low-resource settings [13]. It has been validated previously against the BD FACSCalibur and BD FACSCount in several countries in Eastern and Southern Africa, predominantly in urban reference laboratory or hospital settings, with limited data on performance in rural settings. Its diagnostic accuracy was assessed in a district hospital, an advanced laboratory (Kenya Medical Research Institute/CDC) and in a community level facility in Kenya [8, 19, 21]; at an urban HIV clinic in Johannesburg, South Africa [22], at a hospital-affiliated HIV clinic in Harare, Zimbabwe [20], at a secondary hospital in Nigeria [23] and at four urban facilities in Ethiopia [16]; additionally, a multi-site evaluation took place in hospitals in Kenya, Thailand, San Francisco, and India [14], as well as in reference laboratories in Belgium and Tanzania [15]. Seven out of ten studies reviewed found that Presto over-estimated CD4 count when compared to the reference standard (FACSCount or FACSCalibur). Mean biases ranged from -13.3 cells/μl [16] to +77.16 cells/μl [19]. In addition, all studies comparing venous samples to capillary samples found that capillary samples give a systematically higher estimation of absolute and/or relative CD4 count. Most studies were performed when CD4 count was used to determine ART eligibility, and concluded that the Presto was accurate enough for this application. This study took place before the 2015 “treat all” recommendation was implemented in Ghana, and CD4 count cutoffs were still a critical factor for ART initiation. While all patients are now eligible to begin ART regardless of CD4 count, CD4 remains an important indicator of immune function and access to CD4 testing is still a priority for HIV control programs worldwide.

The aim of this study was to evaluate the accuracy of the Presto in comparison to the BD FACSCount (CD4 Count) and to the Sysmex KX– 21N (hemoglobin concentration) when the Presto was operated in a district hospital and at a rural laboratory in Ghana. To our knowledge, this is the first study of the diagnostic accuracy of Presto in Ghana, one of few in West Africa, and one of few to investigate the feasibility of operating Presto in a limited-resourced, rural laboratory.

## Methods

### Study setting

This diagnostic accuracy study was conducted in two phases in Amansie West, Ashanti Region, Ghana. Phase 1 took place from June to August 2015 at St. Martins District Hospital (DH) in Agroyesum (the only hospital serving Amansie West district), and phase 2 from February to August 2016, in which the Presto was operated in a small rural laboratory in Tontokrom village. Amansie West is one of Ashanti region’s 27 districts and in 2010, had a population of 134,331, 95.5% of whom live in rural areas, and 74.1% subsist off agriculture [24]. The rural laboratory provides basic diagnostic services and serves as a research center. Typical of this level of health care facility, the lab suffers from occasional power outages, water shortages, and limited staff.

At the time of this study, Ghana’s National Guidelines (2010) recommended ART initiation for all PLHIV with CD4 counts less than 300, and for all pregnant women after 14 weeks gestation, regardless of CD4 count. At the same time, WHO recommended ART initiation for PLHIV with CD4 counts of 350 cells/μl or below [25, 26]. Ashanti region had an HIV prevalence of 1.9% in 2014, compared to 2% national prevalence [27]. Ghana formally recommended “treat all” irrespective of CD4 count in October of 2016 (shortly after the conclusion of this study in August of 2016), but Option B+, which recommended lifelong ART for all pregnant women regardless of CD4 count, had not yet been adopted in the region during this study [12, 28].

### Study design

This was a diagnostic accuracy study, separated into two phases, and distinguished by the location of the Presto device either in the district hospital DH (phase 1) or the rural lab (phase 2). In the first phase, all participants were recruited from the DH clinic, and samples were tested on-site. Results from the Presto were compared to the FACSCount (Becton Dickinson, CA, USA) for CD4 Count, and to the Sysmex KX– 21N (Sysmex Corporation, Japan) for hemoglobin, which were also operated at the DH. In the second phase, the Presto was operated in the rural laboratory, and results were compared again to the FACSCount and Sysmex, which were still operated at the DH. Participants in phase 2 were recruited from rural clinics, as well as from the DH. Samples from the DH were therefore transported to the rural laboratory for testing on the Presto. Using Presto at the rural laboratory provided insight into the feasibility of using near-patient CD4 counters in low-resource areas, as compared to the larger and better equipped laboratory at the DH. The Presto test results were never used for patient care in either phase, and all clinical decisions were made based on results from venous blood samples tested on the reference machines.

### Sample collection and testing

Training in the operation of the device, sample collection and basic trouble-shooting, was provided to all participating phlebotomists and technologists by the manufacturer through a one-day training course. During the study, each sample was run on a total of 3 devices: the experimental Presto (for CD4 and hemoglobin), the FACSCount as control for CD4, and the Sysmex as control for Hgb concentration. In phase 1, a capillary blood sample (applied using a lancet to the test cartridge) and a 5 ml aliquot of venous blood from each patient were tested using Presto and the reference machines at the DH HIV clinic. Samples were run on the Presto machine within 30 minutes. All control hemoglobin analyses were performed on the Sysmex on the same day, within a few hours of collection, but control CD4 count analyses were sometimes performed in batches, meaning some samples were stored overnight according to manufacturer’s recommendations and tested the day after collection on the FACSCount.

In phase 2, venous blood was collected at the DH and kept in cold chain at ~8°C, before being transported to the rural laboratory in test tubes by motorbike for testing by Presto the same day. The laboratory is approximately one hour away by motorbike and all samples were tested within 4–5 hours. A second aliquot of blood was tested at the DH on the reference devices (FACSCount and Sysmex). Venous and capillary blood were also both collected from patients at the rural lab, and an aliquot of venous blood was separated into a separate tube and transported by motorbike to the DH laboratory for testing using the control devices; capillary blood was never transported.

### Study population

Adults (≥ 18 years old) from two groups were invited to participate: one group of patients at the DH who came for HIV management and required a CD4 test, and a second group of people from one of the community-level health clinics surrounding rural lab and also required a test. Since chronic HIV care was not yet offered at the majority of community-level clinics (only two out of ten nearby clinics offer ART), only ten participants were recruited from the lower-tier health care facilities. Only participants 18 years or older who gave written consent were included. Subjects included pregnant women but excluded children under 18.

### Sample size

The aim was to collect samples from 100 participants (50 in each phase), but all eligible patients were invited to enroll during the designated study period; 65 patients were recruited to phase 1 and 53 to phase 2. This was a convenience sample and all participants were enrolled consecutively. The sample size was limited by time constraints of the study, and based on normal volume at the district hospital. In phase 2, most participants were recruited from the district hospital, with only ten eligible patients identified and included from the rural health posts; due to low attendance for HIV care at rural clinics at the time. The Clinical and Laboratory Standards Institute (CLSI) recommends at least 40 samples of appropriate range (reflecting both clinical range and analytical range of each method) when conducting accuracy studies [29]; similar studies have used this benchmark to inform decisions on sample size [14, 19].

### Ethics, safety, and informed consent

Eligible participants were informed about the purpose, benefits, and risks of the study, and could ask questions. They gave written consent by circling “Yes” or “No”. When patients were illiterate, the consent form (available in English and Twi) was read aloud. Ethical approval was received from the Institutional Review Board (IRB) at Columbia University (IRB-AAAM4706) and the Ghana Health Service Ethical Review Committee (GHSERC) for both phase 1 and phase 2. Approval from the Ghana Food and Drug Authority (FDA) was obtained for using Presto in Ghana.

### Statistical analyses

Descriptive statistics and measures of association between Presto and the reference methods were calculated using Microsoft Excel, GraphPad Prism, and SAS version 3.5. Two sample T-tests were used to compare mean age of male and female participants, and Wilcoxon Rank Sum tests were used to compare CD4 count between males and females. Three different clinically-relevant cutoff thresholds of CD4+ T Cell count were used: 100 to define the cut-off for use of recommended screening tests for opportunistic infections (OIs) (i.e. TB-LAM and cryptococcal antigen lateral flow assay) in patients with advanced HIV disease; and 350 and 500 cells/μl to reflect past ART initiation recommendations. Rates of upward, downward, and total misclassification, along with sensitivity and specificity for specific cutoffs were calculated to compare performance of the Presto to that of the FACSCount or Sysmex. Wilcoxon Signed Rank tests and Spearman correlation were then used to compare results between methods, using a significance level of 0.05. Bland Altman plots were produced to visualize bias and limits of agreement (LOA) when comparing Presto to FACSCount. Only data from samples which have pair-wise results available was included.

## Results

### Participant characteristics

**Fig 1:**
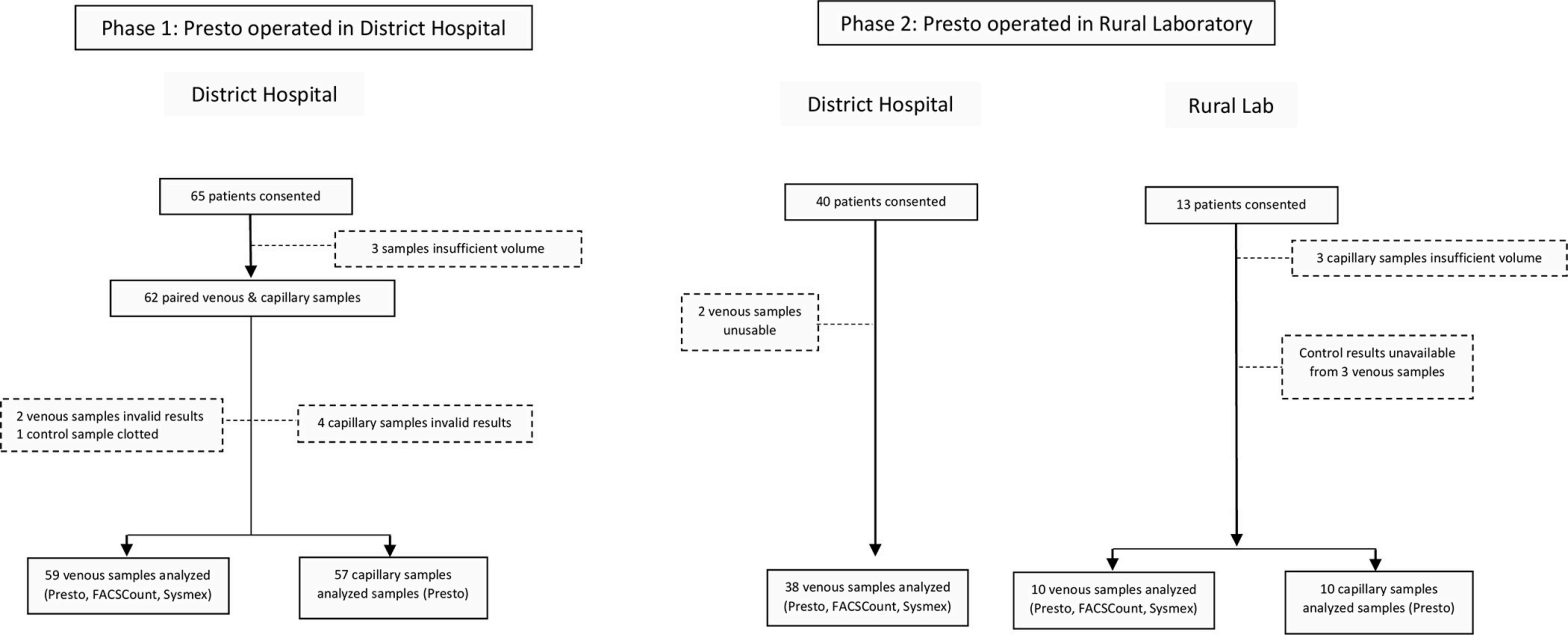
Study recruitment for phase 1 and 2. Participants were recruited sequentially from both the district hospital (DH) and the rural laboratory. In phase 1, all samples were analyzed in the DH from DH patients, and in phase 2, samples came from both DH and rural laboratory patients and all were analyzed in the rural laboratory.

65 patients consented to participate in phase 1, and paired results (Presto and control) from 59 venous samples and 57 capillary blood samples were obtained (Fig 1). In phase 2, 40 patients at the DH consented to participate and venous blood samples from the DH were transported to the rural laboratory, but 2 of these were excluded due to invalid results on Presto. An additional 13 patients from nearby community clinics provided venous and capillary blood samples. Three of the capillary samples were unusable, and control results were unavailable for 3 of the venous samples, resulting in a total of 38 venous samples from the DH and 10 paired venous and capillary samples from the local clinics, representing a total of 48 participants in phase 2. All 48 were tested at the rural laboratory.

The majority (87.3%) of phase 1 study participants were female, with an average age of 40.3 years, of whom 4 were pregnant (Table 1). Men were older than women, with an average age of 53 compared to 40 years (t = 3.55, p<0.001). In phase 2, the majority of participants were again female (62.5%), and the mean age was 41.6 years. Ages were not significantly different between men and women (t = 0.726, p = 0.47). Across both phases, participants were relatively healthy, with only 3.4% (phase 1) and 4.2% (phase 2) of patients with CD4 counts of under 100 cells/μl (according to the FACSCount reference test, Table 2). The majority of participants (61% in phase 1 and 56% phase 2) had CD4 counts above 500 cells/μl. All except one patient from phase 1 reported being on ART, and the patient who had not yet initiated ART had a CD4 count of between 100 and 350 cells/ μl. In phase 1, median CD4 counts appeared higher in women than in men (734 and 478 cells/μl respectively), though only 8 men were present in the phase 1 sample and this was not a statistically significant difference (Wilcoxon rank sum test p = 0.17). In phase 2, median CD4 counts were 650 and 677 cells/μl for women and men, and were not significantly different (p = 0.75). The overall median CD4 count was 595 for patients in phase 1 and 583 for patients in phase 2. Demographic and clinical details are summarized in Tables 1 and 2.

**Table 1:** Demographics: Phase 1 and 2.

	Phase 1				Phase 2			
	Male	Female	Missing	Total	Male	Female	Missing	Total
**n (%)**	8 (12.3%)	57 (87.7%)	0	65	9 (18.8%)	30 (62.5%)	9 (18.8%)	48
**Pregnant**	NA	4 (6.5%)	0	4	N/A	N/A	30	30
**Age Median (IQR)**	51.5 (6.8)	38.5 (12)	n = 1 (1.5%)	43 (16)	43 (8)	39 (18.5)	9 (18.8%)	38 (14.5)
**Age Mean (Min- Max)**	53 (44–70)	40.3 (24–65)	n = 1 (1.5%)	41.9 (24–70)	44.4 (34–58)	41 (19–78)	9 (18.8%)	41.6 (19–78)
**On ART at time of study n (X%)**	8 (100%)	56 (98.2%)	0	64 (98.4%)	N/A	N/A	48 (100%)	N/A

**Table 2:** CD4 count distribution by study phase.

	Phase 1	Phase 2
**CD4 count Median (IQR) (cells/μl), FACSCount**	595 (403–847)	583 (280.5–785.3)
**CD4 ≤ 100 n (%)**	2 (3.4%)	2 (4.2%)
**CD4 > 100 - ≤ 350 n (%)**	8 (14.0%)	10 (20.8%)
**CD4 > 350 - ≤ 500 n (%)**	13 (22.0%)	9 (18.8%)
**CD4 > 500 n (%)**	36 (61.0%)	27 (56.2%)

Note: N/A indicates not available. There were no statistically significant differences between males and females or between the cohort characteristics in phase 1 and phase 2.

### Accuracy

**Fig 2:**
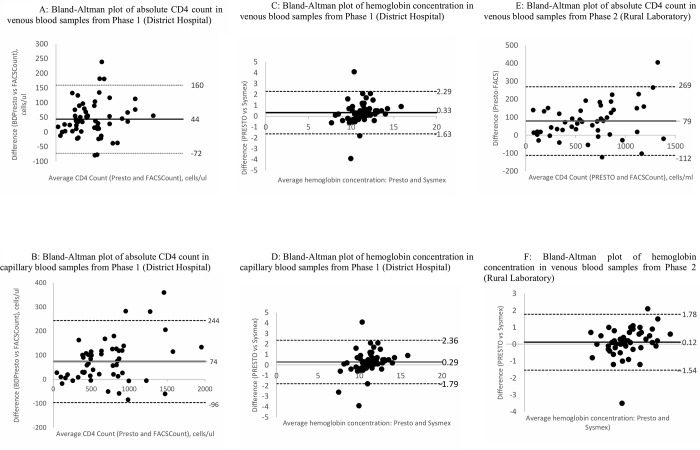
Bland-Altman plots comparing absolute CD4 count and hemoglobin measurements from venous or capillary blood samples as measured with Presto was operated in the district hospital (phase 1) or the rural laboratory (phase 2). Dotted lines indicate the lower and upper limits of agreement and the solid line indicates the bias.

Mean and median CD4 T cell counts for both venous and capillary blood were compared between Presto and FACSCount, at the DH and rural lab. When Presto was operated in the DH (phase 1), higher estimates of both CD4 count and Hgb in venous blood (p<0.001) as well as in capillary blood (p<0.05) were observed. Bland Altman plots were produced to visualize absolute mean bias in CD4 count for samples tested by Presto compared to FACSCount. Bias was 44 (Limits Of Agreement -72, 160) for venous blood, and 74 (LOA -96, 244) for capillary blood (Table 3, Fig 2A and 2B). The Presto also overestimated Hgb concentration with venous (p<0.0007) and capillary blood (p<0.0001) in the DH setting when compared to FACSCount (Table 3, Fig 2C and 2D).

**Table 3:** Summary of venous and capillary samples, bias and correlation between Presto and control devices.

	Phase 1 (DH Lab)					Phase 2 (Rural Lab)				
	Control	Presto-Venous	p	Presto-Capillary	p	Control	Presto-Venous	p	Presto-Cap	p
**Number**	59	59		57		48	48		10	
**CD4 Count (cells/μl)**										
**Mean (Min—Max)**	683 (31–1884)	727.2 (49–1940)		760.12 (59–2018)		596.1 (4–1391)	674.8 (87–1525)		939.3 (589–1480)	
Median (IQR)*	595 (403–847)	651 (422.5–899.5)	<0.001	696 (111–1503)	<0.05	644 (339.75–796.5)	695 (370–924.75)	p<0.001	958 (836–1139)	p = 0.005
**Absolute mean bias (LOA)**	NA	44 (-72, 160)		74 (-96, 244)		NA	79 (-112, 269)		177 (-64.9, 418.3)	
**Relative bias (%), (LOA)**	NA	6.9% (-11.9–25.7%)		10.5% (-11.7–32.7%)		NA	13.1% (-23.7–49.8%)		21.6% (14.5–31%)	
Correlation**		0.98	<0.0001	0.97	<0.0001		0.97	<0.0001	0.93	0.0001
**Hemoglobin Concentration (g/dl)**										
**Mean (Min—Max)**	11.1 (8–15.4)	11.4 (7.4–16.3)		12.2 (8.5–16.2)		11.2 (6.9,15.6)	11.3 (7.0–16.2)		11.4 (8.0–14.2)	
Median (IQR)*	10.9 (10.4–11.8)	11.5 (10.5–12.4)	p<0.0007 (Wilcoxon Signed Rank)	12.3 (11–13.2)	p<0.001	11.1 (10–12.4)	11.0 (9.77–12.65)	p = 0.09	10.7 (10.1–13.5	0.01
**Absolute mean bias (LOA)**	NA	0.3 (-1.6,2.3)		0.3 (-1.79,2.36)		NA	0.12 (-1.54, 1.78)		-.017 (-0.2, 0.1	
**Relative bias (%), (LOA)**	NA	2.3% (-16.9, 9.8%)		1.5% (-20.5,23.5%)		NA	0.5% (-15.7,16.6%)		3.9% (-3.4,11.2%)	
**Correlation** (Spearman’s)		0.74	<0.0001	0.64	<0.0001		0.89	<0.0001	0.88	0.0008

In the rural laboratory setting, the Presto overestimated CD4 counts in both sample types (Table 3, Fig 2E and 2F). Hemoglobin levels were not significantly different for venous blood in the rural laboratory setting (p = 0.09), and were significantly lower for capillary blood tested with the Presto compared to FACSCount (p = 0.01), Table 3.

Spearman’s correlation coefficients demonstrated good correlation for CD4 count (Table 3, all groups at 0.93 or above). For Hgb, correlation coefficients were lower: 0.73, 0.64, 0.89, and 0.87 for venous blood in the district hospital, capillary blood in district hospital, venous blood in the rural laboratory, and capillary blood in the rural laboratory, respectively (Table 3).

When comparing results from venous blood to capillary blood in the DH setting, capillary blood samples gave significantly higher results for both CD4 count and Hgb concentration (Wilcoxon Signed Rank test p = 0.0017 and <0.0001 respectively). Due to a small number of capillary blood samples (n = 10) in the rural laboratory setting, this analysis was restricted to phase 1 data. While capillary blood gave higher CD4 counts overall, Spearman correlation coefficients revealed that CD4 count from capillary and venous blood were highly correlated (Spearman’s Rank Correlation = 0.97, p<0.0001), and correlation between hemoglobin results from capillary and venous blood was lower but still significant (0.80, p < .0001).

Lastly, rates of misclassification, sensitivity and specificity were analyzed across 3 different CD4 count thresholds. There was one instance of downward misclassification, when the Presto classified a sample of venous blood in the rural laboratory as under 350 cells/μl CD4 count, whereas the FACSCount had classified it as above; all other misclassification was upward (Table 4). Accordingly, specificity for all categories was 100% apart from venous blood in phase 2, 350 CD4 count cutoff. Sensitivity was lower, ranging from 50%-100% depending on the CD4 threshold. Only 2 samples were found to have CD4 counts below 100 and sensitivity was poor at this cut-off.

**Table 4:** Data analysis on performance/agreement.

	Venous, District Hospital Lab							Capillary, District Hospital Lab							Venous, Field Lab						
Threshold	Specificity	Sensitivity	PPV	NPV	Up-misclassification, n/N (%)	Down-misclassification, n/N (%)	Total-misclassification, n/N (%)	Specificity	Sensitivity	PPV	NPV	Up-misclassification, n/N (%)	Down-misclassification, n/N (%)	Total-misclassification, n/N (%)	Specificity	Sensitivity	PPV	NPV	Up-misclassification, n/N (%)	Down-misclassification, n/N (%)	Total-misclassification, n/N (%)
100 cells/μl	100.00%	100.00%	100.00%	100.00%	0/2 (0%)	(0/55) 0%	(0/59) 0%	100%	50%	98.21%	100%	(1/2) 50.00%	(0/55) 0%	(1/57) 1.75%	100.00%	50.00%	97.87%	100.00%	(1/2) 50.00%	(0/46) 0%	(1/48) 2.08%
350 cells/μl	100%	70%	94.23%	100%	(3/10) 30.00%	(0/49) 0%	(3/59) 5.08%	100%	70%	94%	100%	(3/10) 30.00%	(0/47) 0%	(3/57) 5.26%	97.22%	91.67%	97.22%	91.67%	(1/12) 8.33%	(1/36) 2.78%	(2/48) 4.17%
500 cells/μl	100%	86.96%	92.31%	100%	(3/23) 13.04%	(0/36) 0%	(3/59) 5.08%	100%	80.95%	90%	100%	(4/21) 19.05%	(0/36) 0%	(4/57) 7.02%	100.00%	76.19%	84.38%	100.00%	(5/21) 23.81%	(0/27) 0%	(5/48) 10.42%

## Discussion

Advanced HIV disease is defined by the WHO as having a CD4 count of below 200 cells/μL, or a disease stage of 3 or 4. Despite advancements in access to ART treatment, 30–40% of PLHIV globally have advanced HIV disease when starting or re-initiating ART [28, 30]. It is critical that access to CD4 testing be assured in HIV programs, even as viral load testing becomes more available [12]. POC CD4 testing was shown to double linkage to care rates in a rural Kenyan setting [5], and same-day blood draw for CD4 testing was shown to significantly increase linkage to care and decrease deferment of care in Durban, South Africa [4]. In addition to impacting linkage to care, assessing the immune status of PLHIV through POC CD4 can identify advanced HIV disease cases, which should be prioritized for prophylaxis for OIs even if they are asymptomatic. A randomized controlled trial conducted in 4 sub-Saharan African countries showed that among patients with CD4 count of below 100 cells/mm^3^, enhanced antimicrobial prophylaxis was associated with reduced rates of death and a large proportion (47%) of patients were either asymptomatic or mildly symptomatic at baseline [31].

Demonstration of device-specific performance can inform the selection of diagnostic technologies for different settings [6]. We evaluated the performance of the near-patient Presto device to test its applicability for measuring CD4 count and Hgb concentration in rural West Africa. Results demonstrated that the Presto consistently over-estimated CD4 count and Hgb concentration when compared to the FACSCount, and that capillary blood samples gave higher estimates than venous blood samples. These results are consistent with findings in other studies. Despite imperfect accuracy, we found that the Presto results for CD4 count and Hgb were well correlated with reference tests FACSCount and Sysmex, and the Presto was successfully operated both in a busy HIV clinic at a district hospital, as well as in a rural laboratory with limited resources. Sample sizes in low CD4 count ranges were insufficient to establish whether many cases of advanced disease would be missed as a result of upward misclassification. In addition, the small number of patients who presented with low CD4 counts meant that upward and downward misclassification rates appear disproportionately high for some groups. For example, one out of two patients with a CD4 count of under 100 was misclassified as being above 100 by capillary sample in the district hospital.

The Presto presents several major advantages, including speed (it has a total test time of approximately 25 minutes, only 4 of which take place in the device), throughput (up to 60 samples in 8 hours), and portability (less than 7 kg and able to be run using battery-power) [12, 13]. On the other hand, it may be important to consider that the Presto gives significant over-estimations of CD4 count and Hgb, and this may result in cases of advanced diseased or anemia being missed. This is particularly relevant for low-level health facilities without venipuncture, since capillary blood demonstrated the lowest accuracy. These biases should be considered by providers when interpreting the results of the Presto, particularly when treating patients with low CD4 count who might be eligible for OI prophylaxis. However, this does not necessarily reduce its suitability and the convenience of a small, near-patient device such as Presto may still greatly improve access to CD4 testing in rural areas as long as this bias is considered.

Like many other West African countries, Ghana has a relatively low HIV burden with an estimated HIV prevalence of 1.6% among adults aged 15 to 49 in 2016, compared to 7% prevalence in Eastern and Southern Africa [32–34]. Control programs in Ghana have worked hard to decentralize HIV services so that access to testing and treatment is adequate throughout the country, even in low-prevalence areas. As Ghana continues to adapt its HIV program and improve rates of early-diagnosis, it is crucial that CD4+ count testing remain accessible for newly diagnosed or ART-initiating patients.

### Limitations

There are several limitations to this study design. Firstly, the sample size is small, with less than 60 patients in each phase. At the time of this study (2015–2016), it was uncommon for HIV patients to seek care at low-level health facilities in Ghana, and only 13 patients were recruited from rural clinics. This resulted in only ten capillary blood samples for this phase of the study, and these were excluded from some analyses due to the small sample size. Overall, CD4 counts were relatively high, so it may not be appropriate to extrapolate these results to patients with low CD4 count.

The technician operating the FACSCount machine at the district hospital (for both phases of the study) was different from the technician operating the Presto. While neither machine requires advanced technical skill, and the same training was provided to all technicians, operator-related differences are possible. Additionally, there are several unknown factors affecting the second phase of the study (rural laboratory). The exact conditions of storage and transport were not systematically documented, and it is possible that the samples were exposed to different temperatures and wait times, potentially impacting the accuracy of the Presto in phase 2. However, as lab testing in resource limited settings often relies on sample transportation to more centralized sites, this scenario may reflect real-world operation of near-patient CD4 testing devices.

Lastly, this study used BD FACSCount as the reference standard, while BD FACSCalibur is normally considered the gold standard among these devices. [8]. However, FACSCount is a widely used device in sub-Saharan Africa and was selected based on its availability, proven accuracy and acceptability in the local setting.

## Conclusion

Reliable CD4 count testing is needed in health facilities to monitor the immune status of PLHIV, even as access to VL monitoring expands. This study found that the Presto overestimates CD4 count in comparison to FACSCount, which is consistent with other studies comparing Presto to FACSCount or FACSCalibur [19–21, 23], though one study in Ethiopia did find a negative bias of 13.3 cells/μl when comparing Presto to FACSCalibur [16]. Overall, the accuracy of the Presto machine must be considered, since over-estimation of CD4 count could result in missing cases of advanced HIV disease, and overestimation of Hgb concentration could result in missed cases of anemia. Future work conducted on much larger datasets could determine whether the bias observed is systematic or random error, and explore possible adjustments to Presto in order to control for overestimation. Despite current limitations however, the Presto can still enable access to CD4 testing, and was successfully operated in a very rural lab setting. It could also be particularly valuable in Preventing Mother To Child Transmission (PMTCT) programs where hemoglobin measurements are clinically important, but further research is needed into the accuracy of hemoglobin measurements from Presto when operated in this context.

## Supporting information

S1 Table BD Presto performance data. This table includes the study ID numbers for all samples analyzed, and the CD4 count and hemoglobin results from BD Presto, BD FACSCount (CD4 Count) and the Sysmex KX– 21N.
(XLSX)
